# Tocochromanol Profiles in *Chlorella sorokiniana*, *Nannochloropsis limnetica* and *Tetraselmis suecica* Confirm the Presence of 11′-α-Tocomonoenol in Cultured Microalgae Independently of Species and Origin

**DOI:** 10.3390/foods11030396

**Published:** 2022-01-29

**Authors:** Alexander Montoya-Arroyo, Katja Lehnert, Alejandra Muñoz-González, Ulrike Schmid-Staiger, Walter Vetter, Jan Frank

**Affiliations:** 1 Department of Food Biofunctionality (140b), Institute of Nutritional Sciences, University of Hohenheim, Garbenstrasse 28, 70599 Stuttgart, Germany; alexander.montoya@nutres.de (A.M.-A.); alejandra.munoz-gonzalez@ucr.ac.cr (A.M.-G.); 2 Department of Food Chemistry (170b), Institute of Food Chemistry, University of Hohenheim, 70599 Stuttgart, Germany; katja.lehnert@uni-hohenheim.de (K.L.); walter.vetter@uni-hohenheim.de (W.V.); 3 School of Food Technology, Universidad de Costa Rica, San Pedro 11501-2060, Costa Rica; 4 Innovation Field Algae Biotechnology-Development, Fraunhofer Institute for Interfacial Engineering and Biotechnology IGB, 70569 Stuttgart, Germany; ulrike.schmid-staiger@igb.fraunhofer.de

**Keywords:** α-tocomonoenol, α-tocopherol, vitamin E, marine-derived tocopherol, tocochromanol biosynthesis, tocotrienols

## Abstract

11′-α-Tocomonoenol (11′-αT1) is structurally related to vitamin E and has been quantified in the microalgae *Tetraselmis* sp. and *Nannochloropsis oceanica*. However, it is not known whether 11′-αT1 is present in other microalgae independent of species and origin. The aim of this study was to analyze the tocochromanol profiles of *Chlorella sorokiniana*, *Nannochloropsis limnetica*, and *Tetraselmis suecica* and to determine if 11′-αT1 is present in these microalgae. Cultured microalgae were freeze-dried and the presence and identity of α-tocomonoenols were confirmed by LC-MS^n^ (liquid chromatography coupled to mass spectroscopy) and GC-MS (gas chromatography coupled to mass spectroscopy). Tocochromanol profiles were determined by HPLC-FLD (liquid chromatography with fluorescence detection) and fatty acid profiles (as fatty acid methyl esters; FAME) by GC-MS. As confirmed by LC-MS^n^ and GC-MS, 11′-αT1 was the dominant αT1 isomer in cultured microalgae instead of 12′-αT1, the isomer also known as marine-derived tocopherol. αT1 represented less than 1% of total tocochromanols in all analyzed samples and tended to be more abundant in microalgae with higher proportions of polyunsaturated fatty acids. In conclusion, our findings confirm that αT1 is not restricted to terrestrial photosynthetic organisms, but can also accumulate in microalgae of different species, with 11′-αT1—and not the marine-derived tocopherol (12′-αT1)—as the predominant αT1 isomer.

## 1. Introduction

Microalgae have been proposed as sustainable food sources to reduce land use competition [1] and as micronutrient-rich alternatives to marine products [2]. Currently, microalgae are used in the production of foods, dietary supplements, and food additives [3,4,5] due to their contents of micronutrients, high-quality protein, bioactive compounds [6], and polyunsaturated fatty acids [7]. *Chlorella sorokiniana* is used for human consumption [8], as feed in aquaculture [9], and as a source of bioactive compounds [10,11]. *Nannochloropsis limnetica* is mainly used in aquaculture [12] and animal feed [13], while *Tetraselmis suecica* is used in aquaculture, as feed, and to prevent bacterial infections [14,15].

Vitamin E comprises a group of eight lipid-soluble compounds (tocopherols and tocotrienols) composed of a chromanol ring attached to a 16-carbon sidechain, collectively referred to as tocochromanols. Based on the number and positions of methyl groups at the chromanol ring, α-, β-, γ-, and δ-congeners of tocopherols (T) and tocotrienols (T3) can be distinguished. T have a saturated side chain and T3 contain a threefold unsaturated side chain [16]. Vitamin E compounds are synthesized by photosynthetic organisms and must be ingested with the diet because of their essential vitamin function in humans [17]. In addition to T and T3, another group of tocochromanols bearing a single double bond in the sidechain has been described and named tocomonoenols (T1) [16].

**Figure 1:**
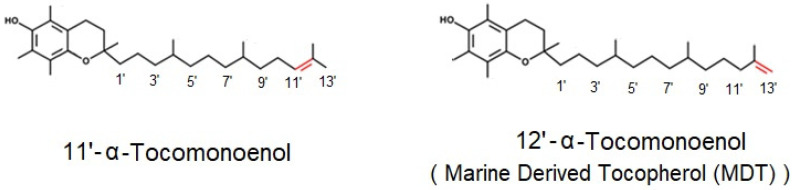
Chemical structure of 11′-α-tocomonoenol (11′-αT1) and 12′-α-tocomonoenol (also known as marine-derived tocopherol (MDT); 12′-αT1) [18,19].

Two different isomers of αT1 (Figure 1) have been reported in the scientific literature, namely 12′-α-tocomonoenol (12′-αT1), originally termed marine-derived tocopherol (MDT) and characterized by a terminal double bond between positions 12′ and 13′ of the alkyl chain [18], and 11′-αT1, with a double bond between carbons 11′ and 12′ [19]. Both αT1 congeners are bioavailable in humans [18], 12′-αT1 has been described as a potential bioactive compound [20], and 11′-αT1 is taken up by liver cells and metabolized similarly to the predominant vitamin E congener αT [21].

12′-αT1 was originally found in marine products, such as fish [18,22,23], fish products [24], and phytoplankton [18,23], while 11′-αT1 has been reported in a range of vegetable oils [25,26,27,28,29] and processed food items containing vegetable oils [30,31]. Although 11′-αT1 has so far been predominantly found in terrestrial plants, we recently reported its presence in cultured cyanobacteria and microalgae sampled from Costa Rica [32].

In *Nannochloropsis oceanica*, 11′-αT1 represented up to 17% of total tocochromanols [32]. Hence, 11′-αT1 may also occur naturally in photosynthetic aquatic organisms and not only in photosynthetic land plants. The aim of the present study was to confirm the presence of 11′-αT1 in the photosynthetic aquatic organisms *Nannochloropsis limnetica*, *Tetraselmis suecica*, and *Chlorella sorokiniana* and to quantify the proportion of 11′-αT1 relative to total tocochromanols in these microalgae. Since 11′-αT1 has been previously reported in the microalgae *Nannochloropsis oceanica*, *Tetraselmis* sp., and *Chlorella vulgaris* [32], we intended with the present study to determine whether this tocochromanol congener has a broader phylogenetic distribution among microalgae.

## 2. Materials and Methods

### 2.1. Microalgae Samples, Culture Conditions, and Sample Pretreatment

*Tetraselmis suecica CCAP66/4* (Chlorodendrophyceae), *Chlorella sorokiniana SAG 211-8k* (Chlorellaceae), and *Nannochloropsis limnetica SAG 18.99* (Eustigmatophyceae) were obtained from the microalgae culture collection SAG (University of Göttingen, Göttingen, Germany) or CCAP, Scotland, UK, and stocked at Fraunhofer IGB (Stuttgart, Germany). All samples were cultured in 6 L commercial flat panel airlift photobioreactors (FPA reactors) using seawater medium for *T. suecica*, DSN media for *C. sorokiniana*, and OHM-media for *N. limnetica*, as described previously [33,34]. The cells were grown to exponential phase, and then the biomass was harvested by centrifugation and freeze-dried (Christ Alpha 1–2 LD freeze drier, Osterode am Harz, Germany), vacuum-sealed, and stored at −20 °C, protected from light and moisture. Samples were ground using an analytical laboratory mill (IKA A11, IKA-Werke GmbH & CO. Staufen, Germany) and stored at −80 °C, protected from light and moisture until analysis. Six independent samples from each microalga were used for analyses.

### 2.2. Standards

Commercial standards of RRR-α-tocopherol, RRR-β-tocopherol, RRR-γ-tocopherol, RRR-δ-tocopherol (purity ≥ 95% Calbiochem-Novabiochem Corp. (Merck Group)), α-tocotrienol, β-tocotrienol, γ-tocotrienol, δ-tocotrienol (purity ≥ 97%, Sigma-Aldrich (Merck Group, Taufkirchen, Germany)), and 11′-αT1 (purity ≥ 97%, isolated from palm oil [29]) were used for tocochromanol identification and quantification. Fatty acids were identified and quantified as fatty acid methyl esters (FAME) using a Supelco 37-Component FAME standard mix (Sigma Aldrich, Taufkirchen, Germany).

### 2.3. Tocochromanol Extraction for Liquid Chromatography

Extraction and quantification of vitamin E congeners were based on Grebenstein et al. [35] with slight modifications, as described elsewhere [32]. In brief, 100 mg of freeze-dried sample was transferred into a glass tube and mixed with 2 mL ascorbic acid 1% (m/v) in ethanol, 90 µL distillated water, and 600 µL saturated potassium hydroxide (KOH) solution. Samples were incubated for 30 min at 70 °C with continuous shaking and then cooled for 5 min on ice. Then, 25 mL BHT solution (1 mg/mL in EtOH), 1 mL distillated water, and 600 µL glacial acetic acid were added and the samples were vortex-mixed. Then, 2 mL of *n*-hexane (HPLC grade) was added and samples were inverted for 1 min. After centrifugation at 280× *g* for 5 min at 4 °C, the supernatant was transferred to a fresh glass tube. The extraction was repeated four times in total. Supernatants (7.5 mL) were pooled and evaporated to dryness (RVC 2-25 CD Plus, Martin Christ Gefriertrocknungsanlagen, Osterode am Harz, Germany). Tocochromanols were resuspended in ethanol, cooled in darkness for 10 min on ice, and centrifuged at 17,000× *g* for 10 min at 4 °C.

### 2.4. Identification of α-Tocomonoenol by Liquid Chromatography Coupled with Mass Spectrometry (LC-MS^n^)

Targeted determination of αT1 isomers was performed as described previously [32]. Briefly, 5 μL tocochromanol extract for liquid chromatography was diluted with 95 μL of pure ethanol, and 5 µL was injected into an Agilent 1290 series HPLC system equipped with a Kinetex PFP column (100 × 4.6 mm i.d., 2.6 μm particle size; Phenomenex, Aschaffenburg, Germany) maintained at 40 °C. The mobile phase was delivered at a flow rate of 0.6 mL/min. Gradient elution with eluent A (methanol/water, 80/20, *v*/*v*) and eluent B (methanol/water, 97/3, *v*/*v*) was applied. The composition of eluent B increased from 0% to 100% within 20 min, was held at 100% B for 5 min, and then returned to 0% eluent B within 2 min and held for 3 min under these conditions. The total run time was 30 min. UV-Vis spectra were recorded with a photodiode array detector within a wavelength range of 190 to 600 nm. The HPLC system was connected to a Q Exactive Plus-Orbitrap mass spectrometer (Thermo Fisher Scientific, Waltham, MA, USA) equipped with an atmospheric pressure chemical ionization (APCI) source operated in positive mode. The scan range (full MS) was set to *m*/*z* 100 to 1000. For MS2 determinations, the lower limit for the scan range was automatically set to *m*/*z* 50.

### 2.5. Identification of α-Tocomonoenol by Gas Chromatography–Mass Spectrometry (GC-MS)

Tocochromanols, including αT1, were extracted and quantified as previously described [36]. In brief, 200 mg of freeze-dried microalgae were extracted three times with 6 mL *n*-hexane and supernatants pooled in an amber glass tube with a screw cap. The solvent was removed under a gentle stream of nitrogen and 2 mL of pyrogallol solution in ethanol (60 g/L), 900 μL demineralized water, and 900 μL KOH solution (50% *m*/*v*) were added. Test tubes were overlaid with nitrogen and incubated with occasional shaking for 60 min at 75 °C in a sand bath. After saponification, samples were cooled on ice and 1 mL demineralized water, 300 μL acetic acid, and 2 mL of *n*-hexane were added, and samples were mixed. Tubes were left for phase separation and the *n*-hexane phase was transferred to a fresh glass tube and washed three times with KOH (pH 9) solution. Then, 500 μL of the *n*-hexane extract were transferred into a 2 mL amber glass vial and evaporated to dryness under a nitrogen stream. Next, 25 mL pyridine (Carl Roth, Karlsruhe, Germany) and 50 μL BSTFA:TCMS 99:1 (Marcherey-Nagel, Düren, Gernamy) were added and samples incubated for 30 min at 70 °C. Once cooled down, solvents were evaporated to dryness under a nitrogen stream and 100 μL of 5α-cholestane (Sigma-Adrich, Taufkirchen, Germany) solution (1.3 μg/mL in *n*-hexane) was used for resuspension. GC-MS runs were performed in full scan mode (mass range: *m*/*z* 50–500) on a 6890/5973 GC-MS system operated in splitless mode (Hewlett-Packard/Agilent, Waldbronn, Germany) and equipped with an OPTIMA 5 TH capillary column (30 m × 0.25 mm i.d., 0.25 μm film thickness) (Marchery Nagel, Düren, Germany). The GC oven program ran 1 min at 55 °C, then it was ramped at 20 °C/min up to 255 °C, then increased at 1.5 °C/min up to 283 °C, then at 15 °C/min until a final temperature of 300 °C that was held for 9 min for a total run time of 39.8 min.

### 2.6. Quantification of Tocochromanols by HPLC-FLD

Clear supernatants (20 μL) of the tocochromanol extract for liquid chromatography were injected into a Jasco HPLC system (controller LC-Net II/ADC, pumps P-U2080 Plus, auto injector AS-2059-SF Plus, column oven co-2060 Plus, mixer LG-2080-02S, degasser DG-2080-53 and fluorescence detector FP-2020 Plus) equipped with a Phenomenex Kinetex PFP column (2.6 μm particle size, 150 × 4.6 mm) maintained at 40 °C, using methanol/water (76/24, *v*/*v*) as the mobile phase at a flow rate of 1.2 mL/min for a total run time of 90 min. The fluorescence detector was operated at excitation and emission wavelengths of 296 and 325 nm, respectively. Peaks were recorded and integrated using ChromNAV software (version 1.19, JASCO, Pfungstadt, Germany) and identified and quantified using 11′-αT1 isolated from palm oil and commercial standards of tocopherols and tocotrienols [32].

### 2.7. Determination of Fatty Acids as Methyl Esters (FAME) by GC-MS

Transesterification of fatty acids was performed as previously reported [32], with minor modifications. Briefly, 4 mg of freeze-dried sample was placed in a glass tube, and 5 μL of internal standard I (DC 11:0; 1 mg/mL) and 1 mL of 1% sulfuric acid in methanol (*v*/*v*) were added and the mixture was incubated at 80 °C for 1 h in a sand bath. During incubation time, the sample was sonicated three times for 5 min in an ultrasonic bath. After incubation, samples were cooled on ice and 1 mL demineralized water, 1 mL saturated NaCl solution, and 2 mL *n*-hexane were added, mixed manually, and then left for phase separation. Then, 5 μL of internal standard II (14:0 EE, 0.5 mg/mL) was added in a vial and mixed with 1 mL of the *n*-hexane supernatant. FAMEs were identified based on retention times and mass spectra in comparison to commercial standards (Sigma Aldrich, Taufkirchen, Germany) using a GC-MS (5890 series II Plus/5972 system with a 7673 autosampler; Hewlett-Packard/Agilent, Waldbronn, Germany) equipped with an Rtx 2330 capillary column (60 m × 0.25 mm i.d. × 0.1 µm 10% cyanopropylphenyl, 90% bis-cyanopropyl polysiloxane; Restek, Bellefonte, PA, USA) in full scan mode. Quantification was carried out in the selected ion monitoring (SIM) mode [37]. The GC oven was heated as follows: after 1 min at 60 °C, the temperature was raised at 6 °C/min up to 150 °C, then at 4 °C/min up to 190 °C, and then at 7 °C/min to a final temperature of 250 °C, which was held for 7 min. Helium (purity 5.0) was used as the carrier gas at a flow rate of 1.2 mL/min [38]. In SIM mode, *m*/*z* 74, *m*/*z* 79, *m*/*z* 81, *m*/*z* 87, *m*/*z* 88, and *m*/*z* 101 were recorded from 7 min (solvent delay) until the end of the run (41.65 min). GC-MS analyses in the full scan mode covered the mass range *m*/*z* 50–500.

### 2.8. Statistical Analysis

Data are reported as arithmetic mean ± standard deviation. The statistical significance of differences in mean values was evaluated using an ANOVA test with a significance level of α = 0.05. Correlation analysis corresponded to Pearson’s correlation test with a significance level of α = 0.05. All statistical analyses were performed using SPSS software (version 22.0, IBM Corporation, Armonk, NY, USA).

## 3. Results and Discussion

We previously reported the presence of αT1 in cyanobacteria (*Arthrospira platensis*) and microalgae (*Nannochloropsis oceanica*, *Tetraselmis* sp. and *Chlorella vulgaris*) collected from tropical zones in Costa Rica. The predominant isomer in all three species was 11′-αT1 [32], even though only 12′-αT1 was previously found in different marine organisms [18,22,23,24]. Therefore, we aimed to determine if 11′-αT1 is present in cultured microalgae from different origins and species, namely *Nannochloropsis limnetica*, *Chlorella sorokiniana*, and *Tetraselmis suecica*, due to their phylogenetic proximity to microalgae previously investigated by our group [32].

### 3.1. Identification of 11′-αT1 and Quantification of Tocochromanols

**Figure 2:**
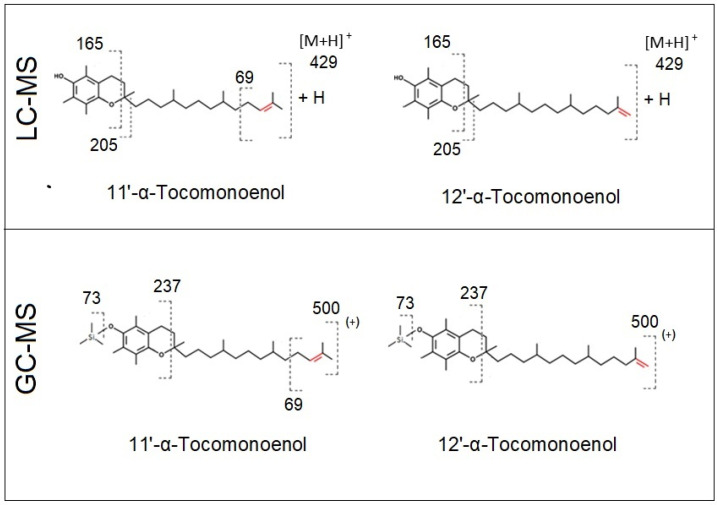
Expected fragmentation patterns of 11′-α-tocomonoenol (11′-αT1) and 12′-α-tocomonoenol (12′-αT1) using LC-MS (upper panel) [40,41] and GC-MS after silylation (lower panel) [29,41].

In order to confirm the presence of 11′-αT1 in *C. sorokiniana*, *N. limnetica*, and *T. suecica*, both LC-MS^n^ and GC-MS approaches were used. The expected fragmentation patterns of 11′-αT1 and 12′-αT1 for both LC-MS and GC-MS are shown in Figure 2. LC-MS^n^ targeted analysis confirmed the presence of αT1 at a retention time of 14.2 min (Table 1) in all analyzed microalgae, based on the characteristic ions *m*/*z* 429.37 ([M + H]^+^) [39], *m*/*z* 205.12, and *m*/*z* 165.09 [29,40]. The occurrence of ion *m*/*z* 69.07 suggested the existence of a double bond between positions 11′ and 12′ [41] and confirms the presence of 11′-αT1 in all the analyzed samples.

**Figure 3:**
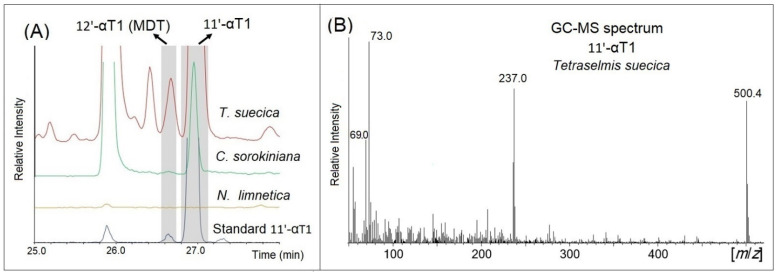
Identification of 11′-α-tocomonoenol (11′-αT1) in *Chlorella sorokiniana*, *Nannochloropsis limnetica*, and *Tetraselmis suecica*. GC-MS chromatograms of *C. sorokiniana*, *N. limnetica*, *T. suecica*, and 11′-α-tocomonoenol (11′-αT1) standard showing the corresponding peaks of 12′-α-tocomonoenol (12′-αT1) and 11′-α-tocomonoenol (11′-αT1) (**A**) and representative GC-MS spectrum obtained for peak 11′-α-tocomonoenol (11′-αT1) in *T. suecica* (**B**).

This is in agreement with results from GC-MS analysis finding a predominant signal corresponding to the retention time and fragmentation pattern of 11′-αT1, not 12′-αT1 (Figure 3A). The corresponding predominant peak at 27.0 min of the GC-MS chromatogram featured the characteristic signals of molecular (*m*/*z* 500) and tropylium ions (*m*/*z* 237) of 11′-αT1 [29] along with *m*/*z* 69, which is associated with a double bond between positions 11′ and 12′ [41] (Figure 3B). This is in agreement with the pattern of isolated 11′-αT1 and was found in all analyzed microalgae samples. These data confirmed and extended our previous findings that 11′-αT1 was present in cultured microalgae, namely *Tetraselmis* sp., *Chlorella vulgaris*, and *Nannochloropsis oceanica* [32].

**Figure 4:**
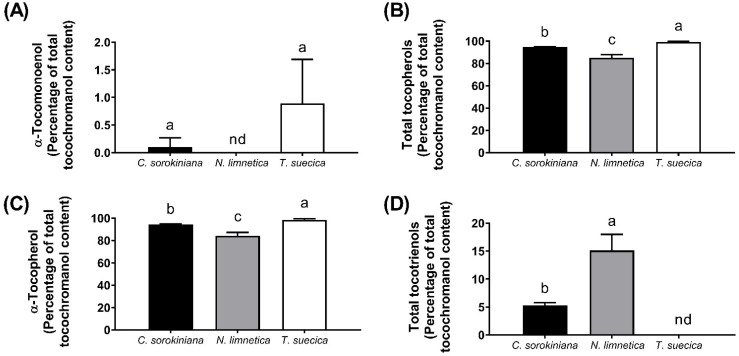
α-Tocomonoenol (αT1) (**A**), total tocopherols (T) (**B**), α-tocopherol (αT) (**C**), and total tocotrienols (T3) (**D**) content expressed as percentage of total tocochromanol content for *Chlorella sorokiniana*, *Nannochloropsis limnetica*, and *Tetraselmis suecica*, as determined by HPLC-FLD. Values are presented as arithmetic mean ± standard deviation (*n* = 6). Bars not sharing a superscript letter are significantly different (ANOVA, α = 0.05). Bars with “nd” refer to congeners not detected by HPLC-FLD.

Tocochromanol profiles of *Chlorella sorokiniana*, *Nannochloropsis limnetica*, and *Tetraselmis suecica* cultured in Germany were obtained by HPLC-FLD and show low but quantifiable amounts of αT1 in *C. sorokiniana* and *T. suecica* (Table 2), corresponding to 0.1% and 0.9% of the total tocochromanol content in these species, respectively (Figure 4A). αT1 was not detected in *N. limnetica* by HPLC-FLD; therefore, its concentration must be below the corresponding limit of quantification (<0.09 mg/kg DW).

These findings are in partial agreement with our previous work that found low but quantifiable concentrations of αT1 in *N. oceanica* (2.5 mg/kg DW, 0.6% of total tocochromanol content), higher concentrations in *Tetraselmis* sp. (15.2 mg/kg DW αT1, 17% of total tocochromanols), and only trace amounts of αT1 in *C. vulgaris* [32]. The observed differences between our current (Table 2) and previous findings [32] show significant variation among species of the same genus. However, absolute comparisons are difficult due to phylogenetic and culture differences among the different species. Significant differences in vitamin E content exist between species of the same genus and have been reported even for microalgae grown under similar culture conditions [42].

The tocochromanol profiles of the microalgae analyzed in the present study were dominated by tocopherols (85% to 99% of the total tocochromanol content; Figure 4B), with αT being predominant and representing 94%, 84%, and 98% of total tocochromanols in *C. sorokiniana*, *N. limnetica*, and *T. suecica*, respectively (Figure 4C). Absolute tocochromanol and tocopherol concentrations in *N. limnetica* were significantly lower compared to other microalgae analyzed in this study (Table 2), but markedly higher compared to previous reports that indicated a total tocopherol content of 21 mg/kg [43]. The total content of tocochromanols in *T. suecica* (654 mg/kg DW) was higher than previously reported, which ranged from 40 to 100 mg/kg DM [44,45,46], but lower than the very high value of 1700 mg αT/kg biomass reported in another study [47]. The αT content in *C. sorokiniana* was higher (682 mg/kg DW) than the ranges (34 to 118 mg/kg DW) reported in the literature [43,48,49].

T3 contents in the analyzed microalgae were low and represented ca. 5% of total tocochromanols in *C. sorokiniana* and 15% in *N. limnetica* (Figure 4D). αT3 was the major T3 in both microalgae, whereas no T3 were found in *T. suecica*. The presence and contents of T3 in microalgae have rarely been reported in the literature and, to the best of our knowledge, have only been confirmed for *C. vulgaris* [32,50], *Tetraselmis* sp., and *N. oceanica* [32]. In agreement with our findings, αT3 was also the major T3 in *C. vulgaris* and *N. oceanica* [32,50]. For the interested reader, additional data regarding the LC-MS-based identification of T3 in microalgae can be found in the Supplementary Materials (Tables S1 and S2). A representative chromatogram for tocochromanol identification in *T. suecica* is presented in Figure S1 in the Supplementary Materials.

### 3.2. Fatty Acid Profiles and Correlations of Fatty Acids with Tocochromanols

Vitamin E is best known for its potent antioxidant activities, i.e., for protecting easily oxidizable substrates, primarily polyunsaturated fatty acids (PUFA), from oxidation in biological membranes (e.g., cell membranes) and food (e.g., vegetable oils) [51]. αT1 was also proposed to protect oxidizable lipids in cold-water fish from oxidation [18,52]. Therefore, we determined fatty acid profiles and quantified PUFA in our microalgae in order to elucidate potential correlations of individual vitamin E congeners and, in particular, αT1 with the content of PUFA.

The vast majority (74–83%) of fatty acids in all three analyzed microalgae were unsaturated fatty acids (Table 3), but significant differences were observed between them. *T. suecica* had the highest content of PUFA (41% of total fatty acids), and 2–3 times more PUFA than *C. sorokiniana* (17%) and *N. limnetica*, (12%). The latter two had 3–4 times higher amounts of di-unsaturated fatty acids (*C. sorokiniana*, 54%; *N. limnetica*, 44%) than *T. suecica* (13%; Table 3). The interested reader is referred to the Supplementary Materials for details on the contents of individual fatty acids (Table S3).

At low temperatures (0 °C), αT1 has been reported to more potently protect liposomal lipids from oxidation than αT and it was therefore proposed that cold-water fish may accumulate αT1 [18]. Similarly, αT is present in particularly high amounts in chloroplasts [53], where it is involved in the protection of PUFA [54] in chloroplast membranes [55]. Hence, αT1 might also confer protection to lipids in aquatic organisms adapted to warmer climates. A higher content of αT1 would then be expected in microalgae with a high content of PUFA, as was indeed observed for *T. suecica* (Table 2). Concentrations of αT1 did not correlate with those of PUFA but a tendency was observed. A significant positive correlation of total tocochromanols was found with omega-3 (ω3) fatty acids (*p* = 0.028), but not with the content of unsaturated, polyunsaturated, monounsaturated, or di-unsaturated fatty acids (Table 4).

The profile of fatty acids and other lipids in microalgae is dependent on the species, but it is also influenced by culture conditions (e.g., light, temperature, and nutrient availability) [56]. Therefore, future studies evaluating the effect of these factors might provide additional information regarding a potential role of αT1 in lipid protection in microalgae.

## 4. Conclusions

In conclusion, we found 11′-αT1 to be the predominant αT1 isomer in cultured *Chlorella sorokiniana*, *Nannochloropsis limnetica*, and *Tetraselmis suecica*. However, αT1 only makes up a small fraction of total tocochromanols (<1%), in contrast to previous reports. We thus confirmed that microalgae are a source of αT1 and that the 11′-αT1 congener is not restricted to terrestrial photosynthetic organisms, but can also be synthesized by microalgae.

## Figures and Tables

**Table 1:** Fragmentation patterns for targeted analysis of α-tocomonoenols (αT1) in *Chlorella sorokiniana*, *Nannochloropsis limnetica*, and *Tetraselmis suecica* using LC-MS^n^.

Sample	RT (min)	Identified Ions
*C. sorokiniana*	14.19	*m*/*z* 429.3729 (C_29_H_49_O_2_; Δ ppm = 0.4) ([M + H]^+^) *m*/*z* 205.1226 (C_13_H_17_O_2_; Δ ppm = 1.6) *m*/*z* 165.0911 (C_10_H_13_O_2_; Δ ppm = 0.4) *m*/*z* 69.0706 (C_5_H_9_; Δ ppm = 11.0)
*N. limnetica*	14.16	*m*/*z* 429.3730 (C_29_H_49_O_2_; Δ ppm = 0.7) ([M + H]^+^) *m*/*z* 205.1224 (C_13_H_17_O_2_; Δ ppm = 0.4) *m*/*z* 165.0912 (C_10_H_13_O_2_; Δ ppm = 1.0) *m*/*z* 69.0707 (C_5_H_9_; Δ ppm = 11.3)
*T. suecica*	14.17	*m*/*z* 429.3727 (C_29_H_49_O_2_; Δ ppm = 0.1) ([M + H]^+^) *m*/*z* 205.1225 (C_13_H_17_O_2_; Δ ppm = 1.1) *m*/*z* 165.0911 (C_10_H_13_O_2_; Δ ppm = 0.6) *m*/*z* 69.0707 (C_5_H_9_; Δ ppm = 11.8)
Standard 11′-α-tocomonoenol	14.24	*m*/*z* 429.3734 (C_29_H_49_O_2_; Δ ppm = 1.6) ([M + H]^+^) *m*/*z* 205.1231 (C_13_H_17_O_2_; Δ ppm = 3.9) *m*/*z* 165.0913 (C_10_H_13_O_2_; Δ ppm = 1.5) *m*/*z* 69.0708 (C_5_H_9_; Δ ppm = 13.4)

**Table 2:** Tocochromanol profiles of *Chlorella sorokiniana*, *Nannochloropsis limnetica*, and *Tetraselmis suecica* determined by HPLC-FLD. Values are arithmetic mean ± standard deviation; *n* = 6. Congeners not sharing a superscript letter are significantly different (ANOVA, α = 0.05).

Congener	Concentration (mg/kg DW)		
	*Chlorella sorokiniana*	*Nannochloropsis limnetica*	*Tetraselmis suecica*
α-Tocopherol	682 ± 9.0 ^a^	70.7 ± 23.03 ^b^	649 ± 86.3 ^a^
β-Tocopherol	0.64 ± 0.07 ^a^	0.23 ± 0.01 ^a^	4.4 ± 0.48 ^b^
γ-Tocopherol	0.05 ± 0.05 ^a^	0.11 ± 0.01 ^ab^	0.16 ± 0.01 ^b^
δ-Tocopherol	0.18 ± 0.02	0.22 ± 0.02	0.4 ± 0.41
α-Tocomonoenol	0.82 ± 1.43	nd	5.5 ± 4.83
α-Tocotrienol	37.4 ± 3.3 ^a^	10.5 ± 1.3 ^b^	nd
β-Tocotrienol	0.02 ± 0.00 ^a^	0.18 ± 0.04 ^b^	nd
γ-Tocotrienol	0.03 ± 0.01 ^a^	1.21 ± 0.09 ^b^	nd
δ-Tocotrienol	nd	0.06 ± 0.00	nd
Total Tocopherols	683 ± 112 ^a^	71.3 ± 23.1 ^b^	654 ± 85.8 ^a^
Total Tocotrienols	37.5 ± 3.3 ^a^	11.9 ± 1.4 ^b^	nd
**Total Tocochromanol**	**721 ± 116** **^a^**	**83.2 ± 24.4** **^b^**	**659 ± 81.0** **^a^**

nd: not detected in the sample.

**Table 3:** Relative fatty acid content (percentage of total fatty acids (g/100 g FA)) in *Chlorella sorokiniana*, *Nannochloropsis limnetica*, and *Tetraselmis suecica* determined by GC-MS as FAME (*n* = 3). Fatty acids not sharing a superscript letter are significantly different (ANOVA, α = 0.05).

Fatty Acid	Relative Fatty Acid Content (g/100 g FA)		
	*Chlorella sorokiniana*	*Nannochloropsis limnetica*	*Tetraselmis suecica*
Monounsaturated	12.7 ± 0.28 ^b^	18.6 ± 0.53 ^a^	20.4 ± 0.04 ^a^
Di-unsaturated	53.5 ± 0.31 ^a^	44.0 ± 0.64 ^b^	12.7 ± 0.10 ^c^
Polyunsaturated	17.0 ± 0.15 ^b^	11.7 ± 0.17 ^c^	40.8 ± 0.24 ^a^
Total Unsaturated	83.2 ± 0.18 ^a^	74.3 ± 0.26 ^b^	74.0 ± 0.24 ^b^
Omega-3	17.0 ± 0.15 ^b^	11.7 ± 0.17 ^c^	25.2 ± 0.42 ^a^
Omega-6	26.0 ± 0.13 ^b^	26.4 ± 0.05 ^a^	15.5 ± 0.09 ^c^
Omega-9	5.4 ± 0.10 ^b^	6.0 ± 0.22 ^b^	10.7 ± 0.03 ^a^

**Table 4:** Pearson correlation coefficient and associated probability (*p*) between vitamin E and fatty acid (FA) content in *Chlorella sorokiniana*, *Nannochloropsis limnetica* and *Tetraselmis suecica*.

Main Variable	Secondary Variable (g/100 g FA)	Pearson Coefficient	*p*
Total tocochromanols (mg/kg DW)	Monounsaturated FA	−0.363	0.337
	Polyunsaturated FA	0.554	0.121
	Total unsaturated FA	0.533	0.139
	Omega-6 FA	−0.441	0.235
	Omega-3 FA	0.722	0.028
Relative tocopherols (%)	Monounsaturated FA	−0.049	0.899
	Polyunsaturated FA	0.795	0.010
	Total unsaturated FA	0.223	0.564
	Omega-6 FA	−0.712	0.031
	Omega-3 FA	0.902	0.001

## Data Availability

The data presented in this study are available on request from the corresponding author.

## Supplementary Materials

The following are available online at https://www.mdpi.com/article/10.3390/foods11030396/s1. Table S1: Fragmentation patterns for commercial standards of tocopherols and tocotrienols using LC-MS^n^. Table S2: LC-MS^n^ fragmentation patterns of tocotrienols in *Chlorella sorokiniana*, *Nannochloropsis limnetica*, and *Tetraselmis suecica*. Table S3: Relative fatty acid content (percentage of total fatty acids) in *Chlorella sorokiniana*, *Nannochloropsis limnetica*, and *Tetraselmis suecica* determined by GC-MS as FAME. Figure S1: Representative chromatogram for tocochromanol identification by HPLC-FLD in *Tetraselmis suecica*.
