# Supplementary material for: Tocochromanol Profiles in Chlorella sorokiniana, Nannochloropsis limnetica and Tetraselmis suecica Confirm the Presence of 11′-α-Tocomonoenol in Cultured Microalgae Independently of Species and Origin
Source: Foods. 2022 Jan 29;11(3):396. doi: 10.3390/foods11030396 (PMC8834470; doi:10.3390/foods11030396)
Supplement: Supplementary file 1 [file foods-11-00396-s001.zip › foods-1556938-supplementary.pdf]

Tocochromanol profiles in *Chlorella sorokiniana, Nannochloropsis limnetica and Tetraselmis suecica* confirm the presence of 11'-α-tocomonoenol in cultured microalgae independently of species and origin.

Alexander Montoya-Arroyo, Katja Lehnert, Alejandra Muñoz-González, Ulrike Schmid-Staiger, Walter Vetter and Jan Frank *

* Correspondence: Department of Food Biofunctionality (140b), Institute of Nutritional Sciences, University of Hohenheim. Garbenstrasse 28, 70599 Stuttgart, Germany. jan.frank@nutres.de, Tel. +49-711-459 24410.

Supplementary Material:

Table S1. Fragmentation patterns for commercial standards of tocopherols and tocotrienols using LC-MS^n.

Sample	Congener	RT (min)	Identified Ions
Tocotrienols	δ-tocotrienol	9.82	*m/z* 397.3102 ($C_{27}H_{41}O_2$; Δ ppm = 0.2) ($[M+H]^+$)<br>*m/z* 177.0910 ($C_{11}H_{13}O_2$; Δ ppm = -0.2)<br>*m/z* 137.0596 ($C_8H_9O_2$; Δ ppm = -1.0)
	β-tocotrienol	10.90	*m/z* 411.3259 ($C_{28}H_{43}O_2$; Δ ppm = 0.3) ($[M+H]^+$)<br>*m/z* 191.1067 ($C_{12}H_{15}O_2$; Δ ppm = 0.0)<br>*m/z* 151.0754 ($C_9H_{11}O_2$; Δ ppm = 0.2)
	γ-tocotrienol	11.40	*m/z* 411.3260 ($C_{28}H_{43}O_2$; Δ ppm = 0.5) ($[M+H]^+$)<br>*m/z* 191.1067 ($C_{12}H_{15}O_2$; Δ ppm = 0.2)<br>*m/z* 151.0754 ($C_9H_{11}O_2$; Δ ppm = 0.2)
	α-tocotrienol	12.32	*m/z* 425.3416 ($C_{29}H_{45}O_2$; Δ ppm = 0.3) ($[M+H]^+$)<br>*m/z* 205.1233 ($C_{13}H_{17}O_2$; Δ ppm = 0.2)<br>*m/z* 165.0911 ($C_{10}H_{13}O_2$; Δ ppm = 0.0)
Tocopherols	δ-tocopherol	12.96	*m/z* 403.3572 ($C_{27}H_{47}O_2$; Δ ppm = 0.5) ($[M+H]^+$)<br>*m/z* 177.0910 ($C_{11}H_{13}O_2$; Δ ppm = 0.0)<br>*m/z* 137.0597 ($C_8H_9O_2$; Δ ppm = 0.0)
	β-tocopherol	13.96	*m/z* 417.3727 ($C_{28}H_{49}O_2$; Δ ppm = 0.1) ($[M+H]^+$)<br>*m/z* 191.1065 ($C_{12}H_{15}O_2$; Δ ppm = -0.6)<br>*m/z* 151.0754 ($C_9H_{11}O_2$; Δ ppm = 0.2)
	γ-tocopherol	14.36	*m/z* 417.3730 $C_{28}H_{49}O_2$; Δ ppm = 0.7) ($[M+H]^+$)<br>*m/z* 191.1065 ($C_{12}H_{15}O_2$; Δ ppm = -0.6)<br>*m/z* 151.0754 ($C_9H_{11}O_2$; Δ ppm = 0.2)
	α-tocopherol	15.21	*m/z* 431.3885 ($C_{29}H_{51}O_2$; Δ ppm = 0.4) ($[M+H]^+$)<br>*m/z* 205.1223 ($C_{13}H_{17}O_2$; Δ ppm = -0.3)<br>*m/z* 165.0910 ($C_{10}H_{13}O_2$; Δ ppm = 0.1)

Table S2. LC-MS^n fragmentation patterns of tocotrienols in *Chlorella sorokiniana, Nannochloropsis limnetica* and *Tetraselmis suecica*.

Sample	Congener	RT (min)	Identified Ions
C. sorokiniana	δ-tocotrienol	9.78	*m/z* 397.3078 ($C_{27}H_{41}O_2$; Δ ppm = -5.9) ($[M+H]^+$)<br>*m/z* 177.0911 ($C_{11}H_{13}O_2$; Δ ppm = 0.6)<br>*m/z* 137.0597 ($C_8H_9O_2$; Δ ppm = -0.1)
	β-tocotrienol	10.80	*m/z* 411.3259 ($C_{28}H_{43}O_2$; Δ ppm = 0.3) ($[M+H]^+$)<br>*m/z* 191.1066 ($C_{12}H_{15}O_2$; Δ ppm = -0.3)<br>*m/z* 151.0754 ($C_9H_{11}O_2$; Δ ppm = 0.0)
	γ-tocotrienol	11.43	*m/z* 411.3257 ($C_{28}H_{43}O_2$; Δ ppm = -0.1) ($[M+H]^+$)<br>*m/z* 191.1430 ($C_{12}H_{15}O_2$; Δ ppm = -0.4)<br>*m/z* 151.0754 ($C_9H_{11}O_2$; Δ ppm = 0.2)
	α-tocotrienol	12.32	*m/z* 425.3409 ($C_{29}H_{45}O_2$; Δ ppm = -2.4) ($[M+H]^+$)<br>*m/z* 205.1223 ($C_{13}H_{17}O_2$; Δ ppm = -2.8)<br>*m/z* 165.0910 ($C_{10}H_{13}O_2$; Δ ppm = -3.2)
N. limnetica	δ-tocotrienol	9.72	*m/z* 397.3095 ($C_{27}H_{41}O_2$; Δ ppm = -1.6) ($[M+H]^+$)<br>*m/z* 177.0911 ($C_{11}H_{13}O_2$; Δ ppm = 0.3)<br>*m/z* 137.0597 ($C_8H_9O_2$; Δ ppm = 0.1)
	β-tocotrienol	10.81	*m/z* 411.3260 ($C_{28}H_{43}O_2$; Δ ppm = -0.8) ($[M+H]^+$)<br>*m/z* 191.1067 ($C_{12}H_{15}O_2$; Δ ppm = -2.8)<br>*m/z* 151.0754 ($C_9H_{11}O_2$; Δ ppm = -3.6)
	γ-tocotrienol	11.43	*m/z* 411.3259 ($C_{28}H_{43}O_2$; Δ ppm = 0.4) ($[M+H]^+$)<br>*m/z* 191.1067 ($C_{12}H_{15}O_2$; Δ ppm = 0.1)<br>*m/z* 151.0754 ($C_9H_{11}O_2$; Δ ppm = 0.0)
	α-tocotrienol	12.30	*m/z* 425.3415 ($C_{29}H_{45}O_2$; Δ ppm = 0.3) ($[M+H]^+$)<br>*m/z* 205.1225 ($C_{13}H_{17}O_2$; Δ ppm = 0.7)<br>*m/z* 165.0911 ($C_{10}H_{13}O_2$; Δ ppm = 0.3)
T suecica	δ-tocotrienol	9.72	*m/z* 397.3095 ($C_{27}H_{41}O_2$; Δ ppm = -1.6) ($[M+H]^+$)<br>*m/z* 177.0910 ($C_{11}H_{13}O_2$; Δ ppm = 0.1)<br>*m/z* 137.0597 ($C_8H_9O_2$; Δ ppm = -0.2)
	β-tocotrienol	10.84	*m/z* 411.3249 ($C_{28}H_{43}O_2$; Δ ppm = -2.0) ($[M+H]^+$)<br>*m/z* 191.1065 ($C_{12}H_{15}O_2$; Δ ppm = -0.8)<br>*m/z* 151.0733 ($C_9H_{11}O_2$; Δ ppm = -0.6)
	γ-tocotrienol	11.43	*m/z* 411.3257 ($C_{28}H_{43}O_2$; Δ ppm = 0.0) ($[M+H]^+$)<br>*m/z* 191.1067 ($C_{12}H_{15}O_2$; Δ ppm = -0.3)<br>*m/z* 151.0753 ($C_9H_{11}O_2$; Δ ppm = -0.4)
	α-tocotrienol	12.33	*m/z* 425.3400 ($C_{29}H_{45}O_2$; Δ ppm = -3.3) ($[M+H]^+$)<br>*m/z* 205.1227 ($C_{13}H_{17}O_2$; Δ ppm = -0.2)<br>*m/z* 165.0910 ($C_{10}H_{13}O_2$; Δ ppm = -0.2)

Table S3. Relative fatty acid content (percentage of total fatty acids, (g/100 g FA)) in *Chlorella sorokiniana, Nannochloropsis limnetica* and *Tetraselmis suecica* determined by GC-MS as FAME (n=3). Fatty acids not sharing a superscript letter are significantly different (ANOVA, α=0.05).

Fatty Acid	Relative fatty acid content (g/100 g FA)		
	Chlorella sorokiniana	***Nannochloropsis limnetica***	***Tetraselmis suecica***
10:0	nd	nd	0.04 ± 0.00
12:0	nd	nd	0.09 ± 0.00
14:0	0.34 ± 0.01 [c]	0.47 ± 0.02 [b]	0.64 ± 0.00 [a]
*i*15:0	0.13 ± 0.00 [b]	0.18 ± 0.01 [a]	nd
*a*15:0	0.01 ± 0.00	nd	nd
15:0	0.04 ± 0.00 [b]	0.06 ± 0.00 [a]	0.03 ± 0.00 [b]
16:0	15.4 ± 0.12 [c]	23.8 ± 0.16 [b]	24.6 ± 0.25 [a]
16:1	5.4 ± 0.1 [a]	3.1 ± 0.13 [b]	1.3 ± 0.05 [c]
16:1*n*-7	1.3 ± 0.05 [c]	8.4 ± 0.06 [a]	2.9 ± 0.04 [b]
16:2	27.3 ± 0.45 [a]	17.6 ± 0.63 [b]	nd
16:3*n*-3	7.0 ± 0.15 [a]	6.1 ± 0.32 [a]	1.4 ± 0.02 [b]
16:4	nd	nd	8.7 ± 0.05
*i*17:0	0.37 ± 0.02 [b]	0.69 ± 0.05 [a]	nd
17:0	0.06 ± 0.01 [b]	0.09 ± 0.01 [a]	nd
17:1	0.19 ± 0.02	nd	nd
17:2	0.20 ± 0.02	nd	nd
18:0	0.42 ± 0.02 [b]	0.43 ± 0.04 [b]	0.63 ± 0.02 [a]
18:1	0.49 ± 0.02 [c]	1.2 ± 0.12 [b]	5.5 ± 0.09 [a]
18:1*n*-9	5.4 ± 0.10 [b]	6.0 ± 0.22 [b]	10.8 ± 0.03 [a]
18:2	0.04 ± 0.01	nd	nd
18:2*n*-6	26.0 ± 0.13 [a]	26.4 ± 0.05 [b]	12.7 ± 0.10 [c]
18:3*n*-3	10.0 ± 0.00 [b]	5.6 ± 0.19 [c]	20.3 ± 0.53 [a]
18:3*n*-6	nd	nd	0.73 ± 0.03
18:4	nd	nd	4.3 ± 0.06
20:4*n*-6	nd	nd	2.1 ± 0.07
20:5*n*-3	nd	nd	3.3 ± 0.11
24:0	0.02 ± 0.00	nd	nd
PUFA*n*-3	nd	nd	0.16 ± 0.02

nd: not detected in the sample.
a: anti-*iso* isomer
i: *iso* isomer

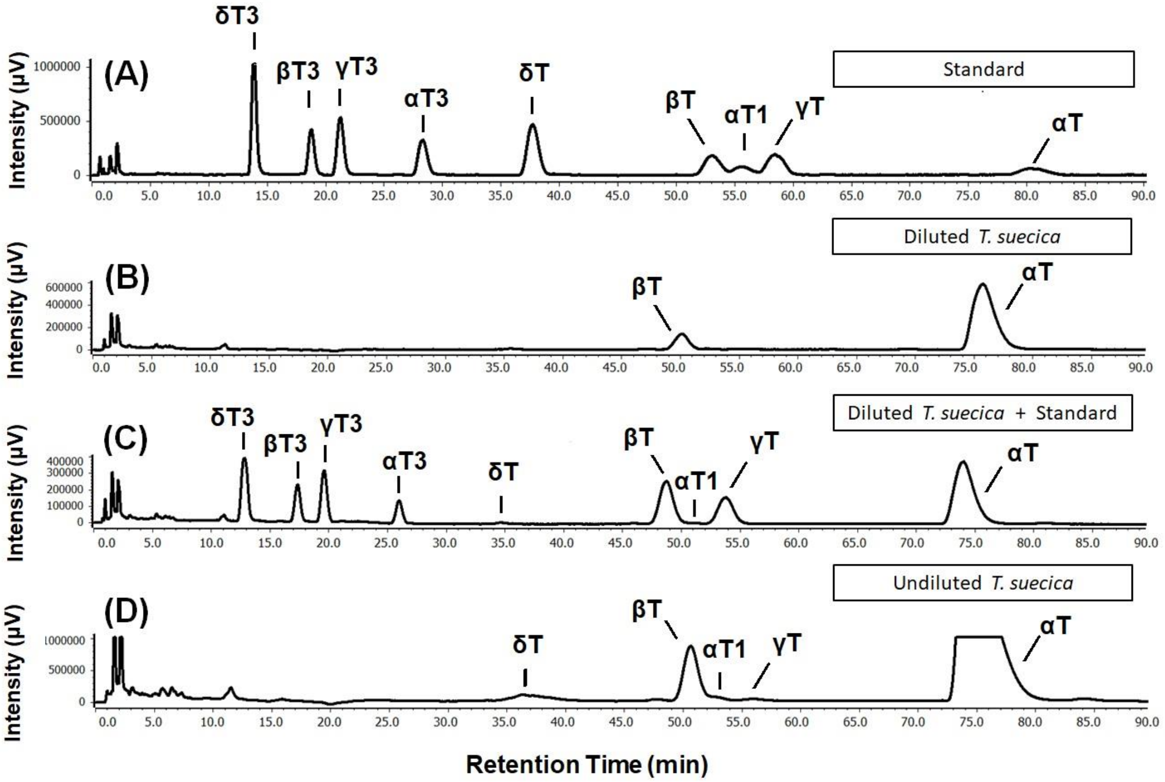

Figure S1. Representative chromatogram for tocochromanol identification by HPLC-FLD. Standard mix of tocotrienols, tocopherols (T), and 11'-α-tocomonoenol (αT1) **(A)**; *Tetraselmis suecica* (sample diluted 1:5 vol/vol with ethanol) **(B)**; *Tetraselmis suecica* (sample diluted 1:5 vol/vol with ethanol) + standard mix **(C)**; undiluted sample of *Tetraselmis suecica* **(D)**.
